# Interplay between stress, sleep, and BDNF in a high-risk sample of young adults

**DOI:** 10.1038/s41598-023-47726-0

**Published:** 2023-11-22

**Authors:** Nimmy Varghese, David Buergin, Cyril Boonmann, Christina Stadler, Marc Schmid, Anne Eckert, Eva Unternaehrer

**Affiliations:** 1 Research Cluster, Molecular & Cognitive Neuroscience, Division of Neurobiology, University of Basel, 4002 Basel, Switzerland; 2 Neurobiology Lab for Brain Aging and Mental Health, Medical Faculty, Psychiatric University Clinics Basel, University of Basel, 4002 Basel, Switzerland; 3 Child and Adolescent Research Department, University Psychiatric Clinics Basel (UPK), University of Basel, Wilhelm Klein-Strasse 27, 4002 Basel, Switzerland; 4 Jacobs Center for Productive Youth Development, University of Zurich, Zurich, Switzerland; 5 LUMC-Curium – Department of Child of Adolescent Psychiatry, Leiden University Medical Center, Leiden, The Netherlands; 6 Department of Psychology, University of Konstanz, Konstanz, Germany

**Keywords:** Sleep, Sleep deprivation, Neurotrophic factors, Stress and resilience, Biomarkers, Risk factors

## Abstract

Children in institutional care have a high risk to experience childhood adversities (CAs), with consequences for physical and mental well-being. The long-term effects of CAs on the brain, including consequences for neuronal plasticity and sleep, are poorly understood. This study examined the interplay between stress (including CAs), sleep, and brain-derived neurotrophic factor (BDNF), a prominent marker for neuronal plasticity. Participants (*N* = 131, mean age = 26.3±3.4 years, 40 females) with residential youth-care history completed questionnaires measuring CAs (Childhood Trauma Questionnaire, CTQ), psychological well-being (World Health Organization-Five Well-Being Index, WHO-5), and sleep disturbances (Pittsburgh Sleep Quality Inventory, PSQI). Hair cortisol and serum BDNF concentration were measured using enzyme-linked immunosorbent assays. The analyses were conducted by using bootstrap regression models. There was no association of stress parameters or sleep with BDNF concentration. However, we found a significant association of CAs and well-being with sleep disturbances. Last, we found an association between CAs and BDNF in sleep-healthy but not sleep-disturbed participants. Our findings indicated a role of sleep disturbance in the association between stress and BDNF. Still, further studies are warranted using vulnerable groups at-risk to understand long-term effects on mental health and sleep.

## Introduction

The human brain is the key organ in stress resilience and vulnerability and acts as a recipient and mediator of stress^1^. The period between early childhood and adolescence is considered one of the most stress-sensitive phases in human development^2,3^. Children in institutional care accumulate multiple biological and psychosocial risk factors towards developing adverse health outcomes, reduced well-being, and psychopathology in adulthood^4–6^. Furthermore, children placed in residential care are likely to experience high levels of childhood adversities and potential traumatic exposures^7^. Childhood adversities (CAs), such as abuse or neglect, are detrimental to neurodevelopment and increase the risk of developing mental disorders^8–10^. However, the relationship between CAs and long-term mental health outcomes is still not fully understood, especially regarding biological parameters beyond the hypothalamic–pituitary–adrenal (HPA-) axis^11^. For instance, brain-derived neurotrophic factor (BDNF) might be a promising biological candidate to investigate the association between CAs and neurodevelopment. Indeed, chronic, or severe early-life stress might adversely impact BDNF levels in the brain^12,13^.

### BDNF

BDNF is one of the highly investigated neuronal growth factors and shows vigorous activity in brain areas necessary for learning, memory, mood, and sleep^14,15^. As a member of the neurotrophic family, BDNF’s role in neurogenesis is defined by differentiation, survival, and synaptic plasticity of neurons^16–18^. BDNF has a high affinity for the tropomyosin receptor kinase B (TrkB), which plays a crucial role in the maturation and regeneration of neurons^14,19^. In addition, BDNF-TrkB binding regulates several downstream pathways, including the protein phosphatidylinositol-3′OH-kinase (PI3K), which in turn activates the AKT-mTOR pathway impacting neuronal survival and apoptosis^14,20,21^. Since neurotrophic factors can cross the blood–brain barrier, serum BDNF concentration (sBDNF) can be detected in peripheral tissue and shows a correlation to brain BDNF levels^22–25^. Therefore, sBDNF may be seen as a justified indicator of neuronal BDNF levels^26^, providing the opportunity to study this promising biomarker.

### Stress and BDNF

The potential clinical relevance of BDNF arises from the evidence of declining BDNF concentrations in numerous psychiatric disorders, aging, and neurodegenerative diseases^14,15,27,28^. Stress experiences could be a trigger in this association. In humans, stress exposure can lead to changes in BDNF expression and thus BDNF levels^29^. The major driving force in the neuroendocrine stress response is the HPA-axis, with its prominent signaling hormone cortisol^30–33^. The release of cortisol regulates response pathways in the body to react to challenging situations, including the secretion of BDNF^29,33^. Via a negative feedback loop in the brain, cortisol regulates its own release^33^. Under acute stress, the finely tuned cortisol response maintains a homeostatic balance. However, prolonged or severe stress, as for example experienced in child trauma, can cause prolonged cortisol exposure, leading to multiple impairments including psychological dysfunction^34–36^. Thus, chronic or severe cortisol exposure plays an adverse role in the BDNF dynamics of neuroplasticity and neurogenesis^37–39^. At the same time, prolonged excessive stress levels might also affect behavioral factors, such as healthy sleep habits, which might in turn impact BDNF levels.

### Sleep and BDNF

In the last decades, the evidence of how sleep quality may interfere with structural plasticity in the brain and thereby affect memory processing is rising^40^. High-quality and sufficient sleep benefits neuronal functioning, including cognitive abilities^41,42^. Sleep supports the learning process immensely by storing and recovering memories^43,44^. Given that sleep disturbances are a common feature in individuals exposed to chronic or severe stress and are associated with aging and psychopathology^23,45–48^. Sleep benefits synaptic plasticity and thereby BDNF seems a good link to investigate the interplay between stress and sleep^49^. Moreover, several links highlight an association between CAs and sleep disturbance^50,51^, but only limited research exists on the long-term effects of CAs on sleep quality in adulthood^52^. Interestingly, a previous study by our group^53^ showed that sleep might be a mediator of the connection between current stress levels and BDNF. Furthermore, sleep might be relevant when considering the association between stress in early life and BDNF^53^.

### The current study

Therefore, the aim of this study was to investigate exposure to adversity in childhood and psychological well-being, cortisol concentration, and sleep disturbances in adulthood as predictors for sBDNF concentration in a high-risk population of young adults with a history of residential youth care. Given our previous findings of an association between insomnia symptoms and sBDNF concentration, we also aimed to replicate our previous findings^53^ in this high-risk population. In line with previous results, we hypothesized that sleep might act as a potential mediator in the association of CAs and psychological well-being with sBDNF. Along with these overall objectives, we investigated the following preregistered hypotheses (https://archive.org/details/osf-registrations-jgs2b-v1): sBDNF concentration is associated with different indicators of stress, such as the severity of CAs (H1a), hair cortisol concentration (H1b), and psychological well-being (H1c). Sleep disturbances are associated with sBDNF concentration (H2a) and indicators of stress, including CAs (H2b), cortisol (H2c), and psychological well-being (H2d). Last, sleep disturbances mediate the association between stress and sBDNF concentration (H3).

For this purpose, data from the “Long-Term Outcomes of Childhood Adversities and Offending Behavior (LOCO)” project was complemented by measuring sBDNF concentration in blood samples collected from these participants^6,54^. All adolescents have been placed in residential youth care. Most have endured various and severe forms of abuse and neglect during childhood and often still suffer from high levels of psychopathology. CAs, psychological well-being, and sleep were assessed using well-established self-report questionnaires (Childhood Trauma Questionnaire^55^, World Health Organization Five Well-Being Index^56^, and Pittsburgh Sleep Quality Index subscale Sleep Disturbance^57^). Biological markers (hair cortisol and sBDNF) were measured using ELISA.

## Methods

### Participants and study procedure

A total sample of 131 young adults with a history of residential youth care participated in this study (91 males, 40 females). These participants were recruited from the “Swiss Study for Clarification and Goal-Attainment in Child Welfare and Juvenile-Justice Institutions” (German: “Modellversuch Abklärung und Zielerreichung in stationären Massnahmen, (MAZ)”); while placed in one of the participating Swiss youth institutional care centers between 2007 and 2012 (for more details see^58–60^). For this study, the participants were reassessed as part of the follow-up “Youth Welfare Trajectories: Learning from Experience” (German: Jugendhilfeverläufe: Aus Erfahrung Lernen (JAEL)) conducted between 2018 and 2020. Participants completed an online survey (www.weaskyou.ch) and a face-to-face assessment, in which several semi-structured clinical interviews were conducted. As a sub-study of the JAEL study, biological material (hair and blood samples) was collected as part of the Long-Term Outcomes of Childhood Adversities and Offending Behavior (LOCO). For more details, see^61–65^). Participant inclusion criteria for this study were participation in the LOCO study, excluding participants with common cold/flue or known immunological diseases (e.g., HIV). Of the 180 JAEL participants on site, 111 provided hair samples (Cortisol) and 131 provided blood samples (BDNF). All JAEL /(LOCO) participants signed an informed consent for participation and received a shopping gift voucher of up to CHF 500. Participant characteristics and a description of continuous study variables are provided in Table 1 (results section). An overall participant flow-chart is provided in the Supplementary Material (Supplementary Fig. 1). We did not find any differences in any of the study variables and covariates between the sample included in the analysis and the sample not included (see Supplementary R Markdown Document on the OSF: https://osf.io/92ktp).

**Table 1:** Participant's characteristics und descriptive statistics of study variables and potential covariates.

	N	Mean	sd	Min	Max
Age	131	26.3	3.5	16.1	38.6
Blood BDNF concentration (ng/ml)	131	28.6	14.4	0.3	103
CTQ total score	130	52.1	15.7	25	101
Hair cortisol concentration (pg/mg)	92	13.3	11.4	1.6	78.2
WHO-5 total score	130	13.7	6	0	25
PSQI sleep disturbance score	130	5.4	4.4	0	20
Number of cigarettes (current smokers only)	78	15.3	8.6	0	50
	*n*	%			
*Sex*					
Males	91	69.5			
Females	40	30.5			
*Childhood socioeconomic status*					
High	59	45.0			
Low	31	23.7			
Missing	41	31.3			
*Migration background*					
Yes	77	58.8			
No	54	41.2			
*BMI (kg/m2)*					
Underweight (BMI < 18.5)	3	2.3			
Normal (18.5 < BMI < 24.9)	58	44.3			
Overweight (25 < BMI < 29.9)	26	19.8			
Obese (BMI > 30)	14	10.7			
Missing	30	22.9			
*SCID-5 positive screening for current insomina*					
Current insomnia	38	29.0			
No current insomnia	93	71.0			
*Chronic medication*					
Yes	75	57.3			
No	43	32.8			
Missing	13	9.9			
	*n*	%			
*Current smokers*					
Yes	78	59.5			
No	15	11.5			
Missing	38	29.0			
*Covariates 2h pior to blood withdrawal*					
Ate food	54	41.2			
Drank caffeine	79	60.3			
Drank alcohol	6	4.6			
Physical activity	15	11.5			
*WHO-5 wellbeing*					
Low	61	46.6			
Normal	69	52.7			
Missing	1	0.8			
PSQI Component Score	Score 0 (*n*)	Score 1 (*n*)	Score 2 (*n*)	Score 3 (*n*)	Missing (*n*)
Subjective sleep quality	14	46	17	7	47
Sleep latency	33	52	25	14	7
Sleep duration	94	16	6	2	13
Habitual sleep efficiency	114	2	2	0	13
Sleep disturbances	11	97	20	2	1
Use of sleep medication	104	8	4	13	2
Daytime dysfunction	38	62	24	5	2
Strain on the CTQ	None (*n*)	Minimal (*n*)	Medium (*n*)	Severe (*n*)	Missing (*n*)
Emotional abuse	59	36	11	24	1
Physical abuse	75	13	19	23	1
Sexual abuse	87	14	13	16	1
Emotional neglect	19	33	25	53	1
Physical neglect	38	31	35	26	1

BDNF = Brain-derived neurotrophic factor; BMI = Body Mass Index; CTQ = Childhood Trauma Questionnaire; max = maximal value; min = minimal value; PSQI = Pittsburgh Sleep Quality Inventory; SCID-5 = Structured Clinical Interview for DSM-5; sd = standard deviation; WHO-5 = World Health Organization Well-being Questionnaire.

### Self-report questionnaires

*CAs* were measured using the Childhood Trauma Questionnaire-Short Version (CTQ-SF)^55^. The CTQ-SF consists of 28 items that measure childhood physical, emotional, and sexual abuse and physical and emotional neglect on a 5-point Likert scale ranging from 1 (never experienced) to 5 (very often experienced).

*Current psychological well-being* was assessed using the World Health Organization-Five Well-Being Index (WHO-5)^56^. The WHO-5 consists of six statements, ranging from 0 (at no time) to 5 (all the time). With a total score of 25, a score lower than 13 indicates a lack of well-being.

Last, *sleep* was assessed using the Pittsburgh Sleep Quality Index (PSQI), which consists of seven subcategories, including sleep disturbances^57^. The questionnaire consists of different response formats depending on the item. For subcategories of sleep disturbances, a 4-point answer scale was used, ranging from 0 (not during the past month) to 3 (three or more times a week). The questions included the following: “During the past month, how often have you had trouble sleeping because you… (1) wake up in the middle of the night or early morning; (2) have to get up to use the bathroom; (3) cannot breathe comfortably; (4) cough or snore loudly; (5) feel too cold; (6) feel too hot; (7) had bad dreams; (8) have pain; (9) other reason(s)”. For this subcategory, sleep disturbances are classified into four component scores (0 = no difficulties, 3 = severe difficulties) based on the sleep disturbances scores summed across nine items (0 total score = 0 component score; 1–9 total score = 1 component score; 10–18 total score = 2 component score; 19–27 total score = 3 component score). Participants were classified as sleep disturbed when scoring above a cut-off score of > 9 total sleep disturbance score (component score of 2 or 3, *n* = 22, Table 1). Although we pre-registered examining the PSQI total score in addition to the sleep disturbance score, we did not analyze the PSQI Total score due to a high number of missing values due to a technical issue (Table 1).

*Potential covariates* were assessed using a sociodemographic questionnaire to measure age and sex. Childhood socio-economic status (SES) was estimated using a 4-point Likert item “Did you have social or financial problems in your family of origin?”. The answers “does not apply” and “does rather not apply” to the question were considered high SES while the answers “does somewhat apply” and “does apply” were considered low SES. Migration background was assessed with a single yes–no item. Biologically relevant covariates – such as medication, Body Mass Index (BMI, measured as weight and height), and cigarette smoking (“do you currently smoke?”, “how many cigarettes do you smoke per day?”), as well as physical activity, food intake, caffeine intake, and alcohol intake less than 2 h prior to blood withdrawal – were assessed using an in-house questionnaire.

### Determination of hair cortisol concentration

The determination of cortisol was done from hair samples collected from the posterior vertex region of the scalp. All participants provided hair of 2 cm adjacent to the scalp, approximately reflecting cortisol secretion over the last eight weeks. The extraction of hair cortisol was done according to^66^. According to the manufacturer's protocols, hair cortisol concentration was measured using the salivary cortisol ELISA kit (Salimetrics Europe, UK). Evaporated samples were resuspended in assay diluent provided by the manufacturer and finally, the absorbance was measured using the Cytation 3 Cell Imaging Multimode Reader (BioTek, USA). The intra-assay coefficient of variation (CV) was 2.3% and the inter-assay CV was 6.1%. Samples were run in duplicates, and mean values of respective concentrations were calculated in pg/mg.

### Assessment of serum BDNF concentration

To assess BDNF levels, a blood sample was collected in the morning (between 8 and 11 am), according to a standardized protocol, using serum Vacutainer tubes (Becton Dickinson). The serum tube was centrifuged at 3000 × g for 10 min. Aliquots were stored at -80 °C prior to the assay. Serum BDNF levels were determined using an enzyme-linked immunosorbent assay (ELISA) kit (Biosensis® Mature BDNF RapidTM ELISA Kit: Human, Mouse, Rat; Thebarton, SA, Australia)^67,68^. Serum samples were appropriately diluted (1:100) and BDNF determination was conducted on a pre-coated mouse monoclonal anti-mature BDNF 96-well plate as described in the manufacturer's protocol. Absorbance was measured within 5 min of the addition of the stop solution in a microplate reader set at 450 nm with a correction wavelength of 690 nm to determine BDNF concentrations according to the standard curve calculated from a 4-parameter logistic curve fit. The intra-assay CV was 2.4% and the inter-assay CV was 8.2% for the BDNF measurements.

### Statistical analysis

The analysis of our study was done in line with our preregistration (10.17605/OSF.IO/JGS2B). In the first step, we determined potential covariates using bivariate correlations between covariates and study variables. Covariates that were significantly associated with the respective dependent variables (H1/H3: BDNF, H2: sleep) were included in the respective statistical models. We then tested our hypotheses using bootstrap regression models, applying 5000 bootstraps resamples. All analyses were conducted using the statistical software R (version 4.2.0), including the packages ggplot2 (version 3.3.6)^69^ and car (version 3.1–0)^70^ for bootstrap regression models. We prespecified that Hypothesis 3 would only be tested if at least one sub-hypothesis of H1 or H2 were confirmed. Analyses were repeated with missing values imputed for independent variables and covariates.

### Ethical approval

This study was conducted in accordance with the Declaration of Helsinki and approved by the Ethics Committee of Northwestern Switzerland (EKNZ; Ref. 2017–00718; 30.06.2017).

### Informed consent

Informed consent was obtained from all subjects involved in the study.

## Results

### Descriptive analysis

All participant characteristics, biological covariates, and descriptive values of study variables are shown in Table 1.

None of the tested potential covariates were significantly associated with serum BDNF concentration or hair cortisol concentration (see Supplementary R Markdown Document: https://osf.io/92ktp). However, women had higher CTQ total scores (*t*(112.26) = −5.12, *p* < 0.001, *d* = −0.81), lower WHO-5 scores (*t*(133.30) = 2.83, *p* = 0.005, *d* = 0.42), and higher PSQI sleep disturbance scores (*t*(115.49) = −3.22, *p* = 0.002, *d* = −0.51) compared to men. Furthermore, we found significant differences in PSQI-SD between high and low SES (*t*(110.41) =  −1.46, *p* = 0.148, *d* = −0.26), between participant with versus without migration status (*t*(160.50) =  −2.30, *p* = 0.023, *d* = -0.35, and between participants taking medication versus those who did not (*t*(82.21) =  −2.93, *p* = 0.004, *d* = −0.55). All correlation coefficients between study variables and potential covariates can be found in the Supplementary R Markdown Document (https://osf.io/92ktp). Assumptions for linear regression models were violated regarding (log)-normal distribution of residuals for H1 and H2, even after transformation or windsorizing of the data. Therefore, we used robust bootstrapped linear regression models, using 5000 bootstrapping resamples for all hypotheses.

Supplementary Table 1 in the Supplementary Materials shows the preliminary bivariate associations between the study variables and tested covariates.

#### Hypothesis 1

Childhood adversity, current cortisol levels and well-being are associated with serum BDNF concentration.

**Figure 1:**
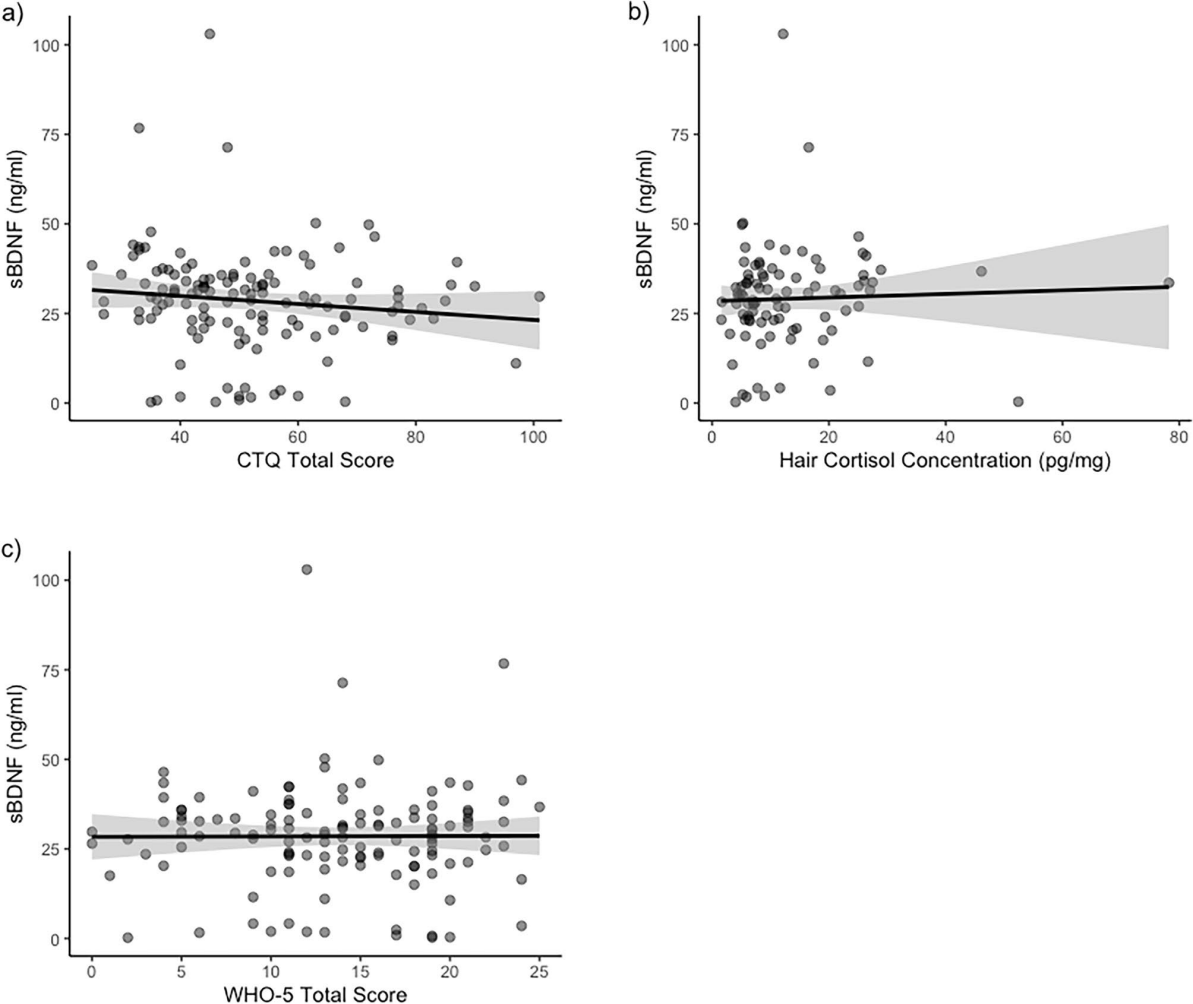
Linear associations of serum BDNF with with (**a**) CTQ Total Score (H1a); (**b**) Hair Cortisol Concentration (H1b); and (**c**) WHO-5 Total Score (H1c). The dots represent the individual subjects, with the darker dots representing overlapping dots. Abbreviations: sBDNF = serum Brain-derived neurotrophic factor; CTQ = Childhood Trauma Questionnaire; WHO-5: World Health Organization Questionnaire.

Because none of the potential covariates was associated with BDNF concentration, we did not adjust the models for any tested covariates according to pre-registration. Despite a significant non-parametric bivariate correlation between CTQ total score and BDNF concentration (Supplementary Table 1), we did not find significant associations between any of the stress indicators (hair cortisol concentration, CTQ total score, WHO-5 score) with BDNF concentration using robust bootstrap regression analysis (Table 2). The associations are illustrated in Fig. 1. Thus, hypothesis 1 was not supported in the data from this high-risk sample. Running the models in the imputed data set did not change interpretation of the results (Supplementary R Markdown Document, https://osf.io/92ktp).

**Table 2:** Results from bootstrap regression analyses for Hypothesis 1 with BDNF concentration as the dependent variable.

Model	Regression parameters					Bootstrap 95% confidence intervals	
	Estimate	SE	Beta	t	p-value	Lower	Upper
Hypothesis 1a (*N* = 130)							
Intercept	34.34	4.39		7.82	< .001	26.85	42.57
CTQ total score	−0.11	0.08	−0.12	−1.38	0.17	−0.25	0.01
Hypothesis 1b (*N* = 92)							
Intercept	28.46	2.32		12.24	< .001	24.43	33.30
Hair Cortisol	0.05	0.13	0.04	0.38	0.71	−0.32	0.27
Hypothesis 1c (*N* = 130)							
Intercept	28.40	3.20		8.86	< .001	22.29	33.79
WHO-5 score	0.01	0.21	< .01	0.05	0.96	−0.34	0.44

bootSE = bootstrapping standard error of regression coefficient; CI = confidence interval; CTQ = Childhood Trauma Questionnaire; WHO-5: World Health Organization Questionnaire.

#### Hypothesis 2

Sleep disturbances are associated with serum BDNF concentration and indicators of stress.

**Figure 2:**
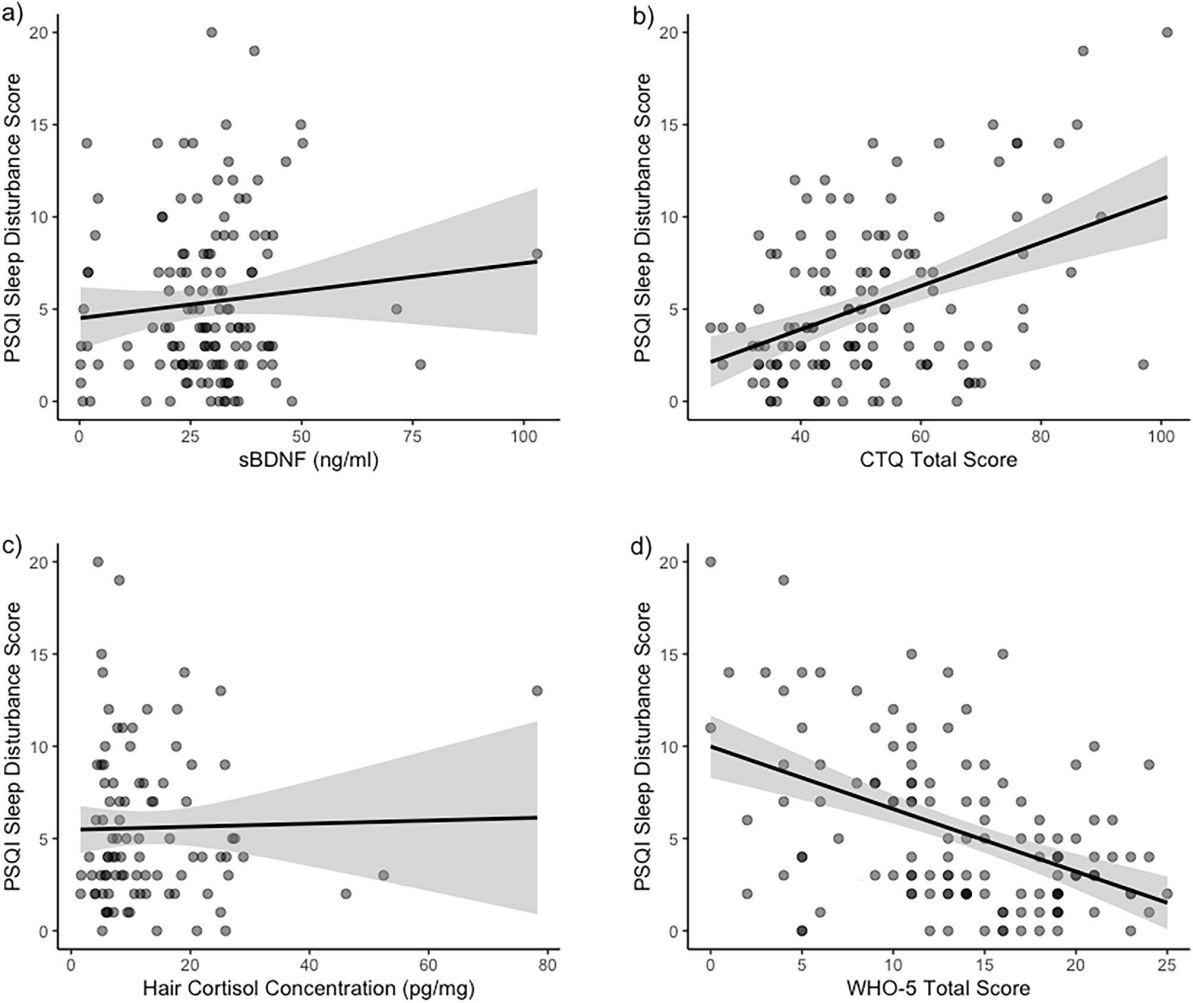
Linear associations of PSQI Sleep Disturbance Score with (**a**) BDNF concentration (H2a); (**b**) CTQ Total Score (H2b); (**c**) hair cortisol concentration (H2c); and d) WHO-5 Total Score (H2d). The dots represent the individual subjects, with the darker dots representing overlapping dots. Abbreviations: BDNF = Brain-derived neurotrophic factor; CTQ = Childhood Trauma Questionnaire; PSQI: Pittsburgh Sleep Quality Inventory; WHO-5: World Health Organization Questionnaire.

We found that sex, socioeconomic status, migration status, and chronic medication were positively associated with PSQI sleep disturbance score (Supplementary Table 1). Therefore, we ran additional analyzes adjusted for these covariates. The associations of BDNF and stress indicators with sleep disturbances are illustrated in Fig. 2.

We did not find support for Hypothesis H2a and H2c: PSQI sleep disturbance scores were neither associated with BDNF concentration nor with cortisol concentration (Table 3). Adjusting for the binary covariates sex, socioeconomic and migration status, and chronic medication did not change the interpretation of these result (Table 3). Age was not controlled for, as it was not correlated with any of the study variables, probably due to the limited age range.

**Table 3:** Results from bootstrap regression analyses for Hypothesis 2 with sleep disturbance as a dependent variable.

Model	Regression parameters					Bootstrap 95% confidence intervals	
	Estimate	SE	beta	t	p-value	Lower	Upper
Hypothesis 2a (*N* = 130)							
Intercept	4.51	0.86		5.27	< .001	3.12	6.28
sBDNF	0.03	0.03	0.10	1.12	0.27	−0.02	0.08
Hypothesis 2a adjusted (*N* = 84)							
Intercept	1.32	1.58		0.84	0.41	−0.99	4.52
sBDNF	0.04	0.04	0.11	1.03	0.31	−0.04	0.11
Sex (male, female)	1.60	1.06	0.16	1.51	0.14	−0.44	3.96
Socioeconomic Status (low, high)	1.88	1.06	0.19	1.77	0.08	−0.04	3.80
Migration Status (no, yes)	1.86	1.05	0.20	1.78	0.08	−0.23	3.71
Medication (no, yes)	1.13	1.04	0.12	1.09	0.28	−1.04	3.06
Hypothesis 2b (N = 129)							
Intercept	−0.81	1.20		−0.67	0.50	−3.52	1.82
**CTQ**	**0.12**	**0.02**	**0.43**	**5.32**	** < .001**	**0.06**	**0.18**
Hypothesis 2b adjusted (N = 83)							
Intercept	−2.97	1.75		−1.69	0.09	−6.72	1.50
**CTQ**	**0.12**	**0.03**	**0.42**	**3.80**	** < .001**	**0.02**	**0.22**
Sex (male, female)	−0.29	1.08	−0.03	−0.27	0.79	−2.84	1.67
Socioeconomic Status (low, high)	1.15	0.99	0.12	1.16	0.25	−0.80	3.13
Migration Status (no, yes)	1.88	0.95	0.20	1.99	0.05	−0.02	3.66
Medication (no, yes)	0.82	0.96	0.09	0.85	0.40	−1.33	2.82
Hypothesis 2c (N = 91)							
Intercept	5.47	0.69		7.91	< .001	4.00	7.11
HCC	0.01	0.04	0.02	0.21	0.83	−0.09	0.10
Hypothesis 2c adjusted (N = 58)							
Intercept	2.11	1.45		1.46	0.15	−0.02	5.34
HCC	0.02	0.05	0.06	0.41	0.68	−0.16	0.11
Sex (male, female)	1.29	1.31	0.13	0.99	0.33	−1.26	4.19
Socioeconomic Status (low, high)	**2.91**	**1.29**	**0.30**	**2.25**	**0.03**	**1.06**	**5.48**
Migration Status (no, yes)	1.44	1.29	0.15	1.12	0.27	−1.39	3.58
Medication (no, yes)	0.41	1.34	0.04	0.31	0.76	−2.09	2.97
Hypothesis 2d (N = 129)							
Intercept	9.99	0.86		11.68	< .001	7.79	12.24
WHO5	**−0.34**	**0.06**	−0.47	**−5.95**	** < .001**	**−0.47**	**−0.21**
Hypothesis 2d adjusted (N = 83)							
Intercept	8.73	1.68		5.20	< .001	4.61	13.74
WHO5	**−0.36**	**0.08**	**−0.47**	**−4.75**	** < .001**	**−0.57**	**−0.18**
Sex (male, female)	0.55	0.96	0.06	0.57	0.57	−1.54	2.90
Socioeconomic Status (low, high)	1.22	0.94	0.12	1.29	0.20	−0.34	3.08
Migration Status (no, yes)	1.03	0.92	0.11	1.11	0.27	−1.09	2.94
Medication (no, yes)	0.83	0.92	0.09	0.90	0.37	−1.38	2.61

BDNF = Brain-derived neurotrophic factor; CI = confidence interval; CTQ = Childhood Trauma Questionnaire; SE = standard error of regression coefficient; WHO-5: World Health Organization Questionnaire.

In contrast, we found support for Hypotheses 2b and 2d: Higher CTQ total score and lower WHO-5 score were associated with higher PSQI sleep disturbance scores and lower WHO-5 score (Table 3). Both predictors remained significant after adjustment for the covariates sex and medication (Table 3). The associations illustrating hypothesis 2 can be found in Fig. 2. Running the models in the imputed data set did not change interpretation of the results (Supplementary R Markdown Document, https://osf.io/92ktp).

#### Hypothesis 3

The association between stress and BDNF concentration are influenced by sleep healthy and sleep disturbed participants.

**Figure 3:**
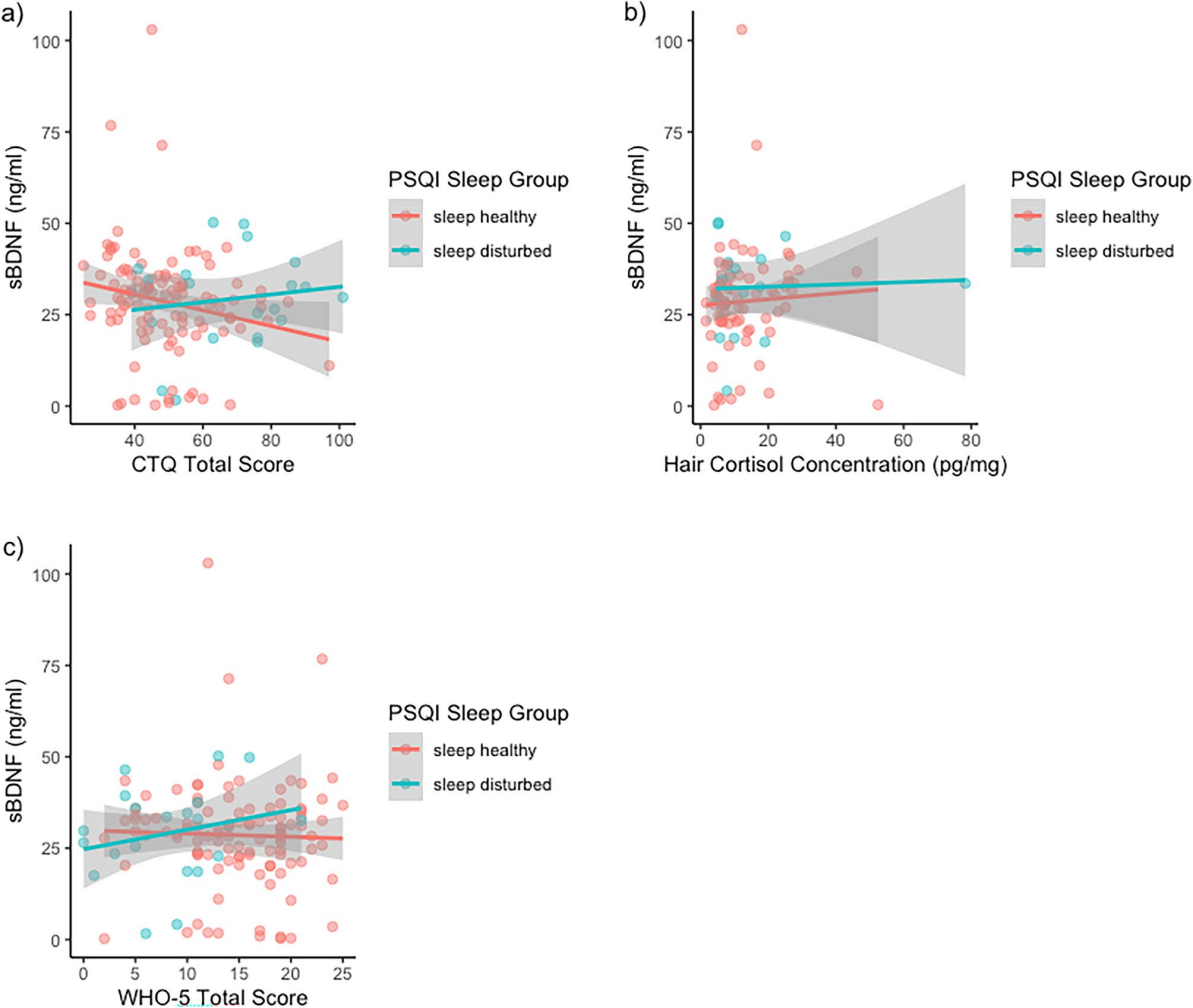
Linear associations by PSQI Sleep Disturbance group between BDNF concentration and (**a**) CTQ total score; (**b**) hair cortisol concentration; and (**c**) WHO-5 score. Abbreviations: BDNF = Brain-derived neurotrophic factor; CTQ = Childhood Trauma Questionnaire; PSQI: Pittsburgh Sleep Quality Inventory; WHO-5: World Health Organization Questionnaire.

Since we did not find an association between stress or sleep and BDNF concentration, and there was also no difference between sleep disturbance groups in BDNF concentration (*t*(33.6) =  −0.362, *p* = 0.720), we did not conduct further mediation analyzes. However, in line with our previous findings, we examined instead the association between BDNF and stress in participants with and without indication of sleep disturbance^53,71^. In line with our previous results, a higher CTQ score was associated with lower BDNF concentration in the “sleep healthy” group (*n* = 108; intercept: *b* = 39.165, *95%-CI* = 30.146 – 48.609; CTQ total score: *b* = −0.216, *boot 95%-CI* = −0.384 – −0.061, *ß* = −0.197), but not in the “sleep disturbed” group (*n* = 21; intercept: *b* = 22.194, *95%-CI* = −2.631– 38.22; CTQ total score: *b* = 0.104, 95%-*CI* = −0.103 – 0.415,* ß* = 0.147). In contrast, when analyzed separately, we did not find any significant associations between BDNF concentration and WHO-5 scores or hair cortisol concentration in any of these two groups. Results are illustrated in Fig. 3 and all parameters are provided in the Supplementary R Markdown Document: https://osf.io/92ktp). Running the models in the imputed data set did not change interpretation of the results (Supplementary R Markdown Document: https://osf.io/92ktp).

## Discussion

The goal of this study was to examine the interplay between stress and sleep regarding BDNF levels in a high-risk sample of young adults with previous residential care placements. Especially children in institutional care are highly prone to adverse health outcomes later in life^72–74^, which makes the investigation of this vulnerable group highly essential. Despite no robust associations of the tested psychological (CAs or well-being) and biological indicators of stress (hair cortisol) or sleep with peripheral BDNF concentration, we found an association between stress during childhood and BDNF concentration in sleep-healthy adolescents and adults, but not in those suffering from sleep disturbances, replicating our previous findings.

### Association between stress and BDNF

Despite a significant non-parametric association between CTQ and BDNF concentration, we did not observe a robust link between the CTQ score or any of the concomitant stress parameters and serum BDNF across the entire sample. Thus, data only provided weak support for Hypothesis 1a (association between CAs and BDNF) and no support for Hypothesis 1b (association between cortisol and BDNF) or 1c (association between psychological well-being and BDNF). Our inconsistent findings align with previous studies investigating CAs and BDNF with contradicting results, specifically by indicating both, BDNF increases^75,76^ or declines^76,77^ in individuals with a history of CAs. In a systematic review^78^ comparing twenty-one studies, no significant differences were found in BDNF concentration between CAs-exposed and non-exposed groups. The review furthermore revealed that the correlation between CAs and BDNF might depend on age. Specifically, studies in children and adolescents younger than twenty years found higher BDNF levels after exposure to CAs, while the correlation diminished later in life. This elevated BDNF level was discussed to be a compensatory mechanism to counteract the stress-related impairment in the brain of young adults^79^. This compensation may be dampened in matured adulthood, leading to a higher vulnerability to develop psychological disorders by maintaining a stressful environment^80,81^. Given that the association between ELA and BDNF might be age dependent and that the age range in this study spanned from adolescence to adulthood (age range 16 – 38 years), we speculate that a potential association might have been masked in our age-diverse sample.

In addition, the association between stress and BDNF might depend on whether someone suffers from sleep disturbances or not^53,71^.

### Association of sleep with BDNF and stress

Despite the apparent role of sleep in the association between stress and BDNF, sleep itself might impact BDNF concentration^53,71,82^. Therefore, we also examined the direct link between sleep and BDNF concentration (Hypothesis 2a). Although several studies indicated a relationship between good sleep and higher BDNF levels^71,82^, we observed no association between sleep disturbances and serum BDNF concentration. This might be due to the specific risk-profile of our sample. Nevertheless, in line with our expectations and a rich body of literature, we found a significant association between sleep disturbances and participant-reported stress, including CAs (Hypothesis 2b) and current well-being (Hypothesis 2d). These findings further support the health concerns caused by sleep disturbance^83^, potentially due to higher levels of perceived stress^84^. In particular, our findings indicate a long-term effect of CAs experiences on sleep quality, potentially mediated by lower levels of well-being (WHO-5). Sleep disturbances are often related to increased susceptibility to stress, making them a common feature in the pathogenesis of stress-related disorders^85^. Thus, our results highlight the importance of subjective indicators of perceived stress and well-being in relation to sleep, thereby replicating this known association in a rare high-risk sample of residential care leavers^86–88^. In contrast to subjective reports of CAs and well-being, there was no link between hair cortisol concentration (an objective indicator of stress) and sleep disturbances (Hypothesis 2c). However, previous studies have yielded inconsistent findings regarding this association^89^. Our finding suggests that changes in HPA axis activity did not underly the association between CAs and sleep disturbances. Another variable to consider is the potential variation in the prevalence of sleep disturbance among our sample of individuals who have experienced a high level of childhood adversity compared to the general population. According to previous research, the prevalence of sleep problems in the general population varies between 20 to 41.7%^90^, while Western Europe reports a prevalence of 31%^91^. The observed prevalence of 30%—as determined by a screening question on the Structured Clinical Interview for DSM-5 (SCID-5^92^) aligns with the global range of prevalence. Nevertheless, the PSQI score indicates a marginally decreased prevalence of sleep disruption by 17%. The lower prevalence observed in our sample could be attributed to our small sample size or the younger age of participants (Age_mean_ = 26.3 years).

### Secondary findings

As our previous analyses did not reveal a significant relationship between the different stress parameters or sleep and BDNF in the entire sample, it was unlikely that sleep disturbances would have an indirect effect or even mediate a relationship between stress and BDNF. Therefore, according to our previous study, we examined the association between stress and BDNF stratified by sleep disturbances: in sleep healthy subjects and those suffering from sleep disturbances. Indeed, our findings were consistent with our previous study^53^, which demonstrated an association between perceived chronic stress and BDNF concentration only in those who did not suffer from insomnia. Therefore, we extend previous findings by showing similar effects when considering CAs instead of current perceived stress levels. Comparing to our previous study, it must be pointed out that different sleep quality parameters were accessed in the two studies: Insomnia Severity Index (ISI) and PSQI. However, several groups comparing the ISI and PSQI demonstrated a moderate to strong correlation between these sleep indicators^93,94^. Therefore, our data underscore our earlier key findings regarding a complex interplay between stress, sleep, and BDNF.

### Clinical relevance

BDNF administration has a potential therapeutic capability to counteract neurodegeneration as an antidepressant tool^14,19,95^. From our results, sleep quality is an additional parameter to consider in a potential therapeutic approach against stress-induced depression. Others have shown that insufficient sleep triggers depression by affecting neuronal circuitries^96^. Sleep is essential in regulating neuronal differentiation and -survival, whereby chronic sleep disturbance may impair the BDNF circuitry in response to stress^97,98^. From the perspective that children in institutional care acquire biological and mental risk factors towards developing psychopathology, improving sleep might constitute an effective and economical way to counteract some of the adverse effects of CAs and their conveyed risk for depression, thereby enhancing well-being^99^.

This study investigated a vulnerable population accumulating multiple biological and psychosocial risk factors leading to severe mental health impairment later in life^6,73,74^. Using this high-risk sample is essential in investigating potential triggers and biological markers for psychopathology. Despite this strength, a high-risk sample also conveys some limitations. One concern is the cross-sectional nature of our data. A longitudinal study design with multiple biological sampling timepoints after adversities and using lagged models might help a more causal understanding of the interplay between CAs, sleep, stress, and BDNF. Especially the long-term tracking of stress, sleep quality, and BDNF concentration in the context of CAs would be promising in disentangling potential links between these variables. This may be the reason why we did not find an association between the different stress indexes and BDNF. A time-distal measure of BDNF has the risk for a diminished impact of CAs on this biomarker, particularly considering age-dependent findings. The age dependence also highlights the importance of the sample collection timing based on participant age yielding more homogenous age groups when using BDNF as a quantifiable biomarker for CAs. In this way, the biological pathways through which CAs negatively impact physical and mental health may be elucidated in greater depth by future studies.

A major concern regarding our data set was the large number of missing values in the PSQI questionnaire (particularly on the subjective sleep quality component) and the following inability to calculate a reliable PSQI total score for the majority of participations. As we pre-registered our variables prior to examination of the data, we were unaware of this large number of missing values. Therefore, we choose to focus on the PSQI sleep disturbance component as described in the pre-registration to preserve the credibility of our findings. We argue that the main issue with this questionnaire was not the non-compliance of the study participants, but rather a technical problem: Some questions had an unfortunate answer format that was unsuited for completion on a tablet device. In contrast to the subjective sleep quality component, the sleep disturbance component questions were answered by all but one participant, making the use of the sleep disturbance score more reliable. This score consisted of items on physical discomfort during bedtime such as feeling too hot or too cold, breathing problems, bad dreams, pain, coughing, snoring, and bathroom usage during the night. However, it should be noted that experiencing one or two of these parameters alone is not enough to indicate impaired sleep, as even healthy individuals may experience them from time to time. Furthermore, the accuracy of the PSQI was validated using one insomnia screening question on a conducted semi-structured clinical interview (Structured Clinical Interview for DSM-5; SCID-5, first^92^). Overall, there was a positive association between the SCID5 Insomnia Screening question and the PSQI sleep disruption category (W = 1091.5; *p* < 0.001). Furthermore, when the insomnia screening question was used to stratify the participants into a sleep healthy and a sleep disturbed group, all results from hypothesis 3 were replicated.

The present study's limitations also include the participants low recruitment efficacy. First, residential and foster care placements present instability in the long-term, with adverse psychological, social, and behavioral factors making recruitment difficult^100^. Thus, the small sample size in the current investigation might underpower our analysis considering the small effect sizes. Second, another limitation linked to our sample pool is the homogeneity of the subjects regarding a high psychosocial burden in these at-risk young adults, which could dampen any potential effects affecting the interplay between biomarkers and psychological and behavioral parameters. Third, the sample consisted of young adults with high levels of childhood adversity, which might still cause substantial burden as confirmed by a significant association between CTQ and WHO-5. Interestingly, it seemed that it was the more the distal stress experience (CA) that was related to BDNF concentration, rather than the more proximal stress measures, such that results for hypothesis 3 remained stable even when controlling WHO-5 or HCC, suggesting that potential molecular programing mechanisms might be at play. Therefore, future studies could examine the association between CA and patterns in *BDNF* DNA methylation. Fourth, the lack of a control group without residential care compromises the comparability of our study. Hereby, an unbiased control is a potential factor to tackle the variance constraints and increase our sample dimension. Fifth, although the estimated prevalence of posttraumatic stress disorder was low, we were not able to make in-depth comparisons between those suffering from PTSD and those without PTSD. Nevertheless, additional analyzes revealed that sBDNF, HCC, and WHO-5 score were not associated with PTSD while PSQI sleep disturbance was related to PTSD (data not shown). However, including PTSD as a potential covariate did not change interpretation of any results. Last, due to the need for more parameters in the self-reports, comparisons between several key indicators could not be conducted. For example, it would be interesting to investigate other variables of well-being, resilience, and health outcomes.

Despite these shortcomings, this study provides rare insight into a well-phenotype high-risk population comparing CAs, sleep disturbance and serum BDNF levels. The opportunity to access biological parameters in young adults with previous residential care placements is rare, making our findings more valuable as they provide a glimpse at this vulnerable population. With our analysis in this high-risk group, we could replicate and strengthen our previous findings and thereby show the importance of considering sleep quality in the association between BDNF and stress in a high-risk sample with a history of youth care placement.

Our study using a highly burdened subgroup of residential care leavers allows us to understand the long-term effects of CAs on the brain to overcome potential deficits leading to psychological diseases, such as depression^101–103^. At the same time, this highly specific group limits the generalizability of our findings. Nevertheless, the lack of available research on CAs and neuronal molecules, such as BDNF, is a fundamental issue^78^. Therefore, our findings provide a small contribution to gain insights into CAs' long-term effects on BDNF for future researchers.

## Conclusion

In summary, this study investigated the interplay between sleep, stress and BDNF in a high-risk sample of young adults with a history of residential care placement. Although we did not find a robust association of CAs, psychological well-being, hair cortisol concentration, or sleep with sBDNF concentration in the entire sample, we found that subjects without sleep disturbances showed an association between CAs and sBDNF concentration. We further demonstrated an association between stress and sleep quality, replicating previous findings in a high-risk sample. While our evaluation suggests that sleep disturbance plays a critical role in the relationship between stress and BDNF, further clinical studies, especially in highly diverse groups, are necessary to validate our findings and thoroughly explore the complex interplay between these factors. Furthermore, it is essential to clarify the role of BDNF as a neuronal modulator in this interplay. Despite limitations, the presented findings are valuable considering the importance of sleep quality on the perception and adaptation of stress. Overall, our findings contribute to a growing body of evidence suggesting sleep impairment as a crucial factor when considering a potential association between stress and BDNF, thereby supporting our previous study^53^. Further studies are warranted to increase our understanding of how stress, sleep, and sBDNF concentration play together to shape health and well-being. Moreover, the inclusion of highly vulnerable participants with a high psychosocial risk complements previous studies in community and clinical populations. Particularly in high-risk populations, the long-term consequences and severity of trauma and other adverse stress events must be taken into account to improve, prevent, and counteract the risks of adversities for young adults.

## Supplementary materials

The following supporting information can be downloaded from the OSF project at https://osf.io/erw24/.

### Supplementary Information

Supplementary Information.

## Data Availability

Data is available upon request from the corresponding author (E.U.).

## References

[1] McEwen BS (2007). Physiology and neurobiology of stress and adaptation: central role of the brain. Physiol. Rev..

[2] Humphreys KL (2019). Evidence for a sensitive period in the effects of early life stress on hippocampal volume. Dev. Sci..

[3] Heim C, Binder EB (2012). Current research trends in early life stress and depression: review of human studies on sensitive periods, gene-environment interactions, and epigenetics. Exp. Neurol..

[4] Gander T (2019). Predictive factors for changes in quality of life among children and adolescents in youth welfare institutions. Soc. Psychiatry Psychiatr. Epidemiol..

[5] Garcia AR (2017). Adverse childhood experiences among youth reported to child welfare: Results from the national survey of child & adolescent wellbeing. Child Abuse Negl.

[6] Burgin D (2022). Adverse and traumatic exposures, posttraumatic stress disorder, telomere length, and hair cortisol - Exploring associations in a high-risk sample of young adult residential care leavers. Brain Behav. Immun. Health.

[7] Bellis MA (2019). Life course health consequences and associated annual costs of adverse childhood experiences across Europe and North America: a systematic review and meta-analysis. Lancet Public Health.

[8] Green JG (2010). Childhood adversities and adult psychiatric disorders in the national comorbidity survey replication I: associations with first onset of DSM-IV disorders. Arch. Gen. Psychiatry.

[9] Enns MW, Cox BJ, Clara I (2002). Parental bonding and adult psychopathology: results from the US National Comorbidity Survey. Psychol. Med..

[10] Danese A (2009). Adverse childhood experiences and adult risk factors for age-related disease: depression, inflammation, and clustering of metabolic risk markers. Arch. Pediatr. Adolesc. Med..

[11] Dempster KS (2021). Linking the hemodynamic consequences of adverse childhood experiences to an altered HPA axis and acute stress response. Brain Behav. Immun..

[12] Aas M (2019). Reduced brain-derived neurotrophic factor is associated with childhood trauma experiences and number of depressive episodes in severe mental disorders. Schizophr Res..

[13] Watt T (2020). The unique nature of depression and anxiety among college students with adverse childhood experiences. J. Child Adolesc. Trauma.

[14] Bjorkholm C, Monteggia LM (2016). BDNF - a key transducer of antidepressant effects. Neuropharmacology.

[15] Colucci-D'Amato, L., L. Speranza, and F. Volpicelli, Neurotrophic factor bdnf, physiological functions and therapeutic potential in depression, neurodegeneration and brain cancer. Int. J. Mol. Sci., 2020. **21**(20). 10.3390/ijms21207777 PMC7589016 33096634

[16] Nordvall G, Forsell P, Sandin J (2022). Neurotrophin-targeted therapeutics: a gateway to cognition and more?. Drug Discov. Today.

[17] Leal G, Comprido D, Duarte CB (2014). BDNF-induced local protein synthesis and synaptic plasticity. Neuropharmacology.

[18] Lu B, Nagappan G, Lu Y (2014). BDNF and synaptic plasticity, cognitive function, and dysfunction. Handb Exp. Pharmacol..

[19] Allen SJ (2013). GDNF, NGF and BDNF as therapeutic options for neurodegeneration. Pharmacol. Ther..

[20] Bai J (2022). Exercise facilitates the M1-to-M2 polarization of microglia by enhancing autophagy via the BDNF/AKT/mTOR pathway in neuropathic pain. Pain Physician.

[21] Zhu JX (2019). Gallic acid activates hippocampal BDNF-Akt-mTOR signaling in chronic mild stress. Metab Brain Dis..

[22] Klein AB (2011). Blood BDNF concentrations reflect brain-tissue BDNF levels across species. Int. J. Neuropsychopharmacol..

[23] Schmitt K, Holsboer-Trachsler E, Eckert A (2016). BDNF in sleep, insomnia, and sleep deprivation. Ann. Med..

[24] Binder DK, Scharfman HE (2004). Brain-derived neurotrophic factor. Growth Factors.

[25] Wolf, I.A., Effekte von Stress, sozialer Unterstützung und Persönlichkeitsvariablen auf psychisches Befinden*.* 2008.

[26] Sartorius A (2009). Correlations and discrepancies between serum and brain tissue levels of neurotrophins after electroconvulsive treatment in rats. Pharmacopsychiatry.

[27] Erickson KI, Miller DL, Roecklein KA (2012). The aging hippocampus: interactions between exercise, depression, and BDNF. Neuroscientist.

[28] Rana T (2021). Unfolding the role of BDNF as a biomarker for treatment of depression. J. Mol. Neurosci..

[29] Linz R (2019). Acute psychosocial stress increases serum BDNF levels: an antagonistic relation to cortisol but no group differences after mental training. Neuropsychopharmacology.

[30] Juruena MF (2020). The role of early life stress in HPA axis and anxiety. Adv. Exp. Med. Biol..

[31] Leistner C, Menke A (2020). Hypothalamic-pituitary-adrenal axis and stress. Handb Clin. Neurol..

[32] Heim C (2008). The link between childhood trauma and depression: insights from HPA axis studies in humans. Psychoneuroendocrinology.

[33] Dedovic K (2009). The brain and the stress axis: the neural correlates of cortisol regulation in response to stress. Neuroimage.

[34] Jankord R, Herman JP (2008). Limbic regulation of hypothalamo-pituitary-adrenocortical function during acute and chronic stress. Ann N Y Acad Sci.

[35] Harrewijn A (2020). Associations between brain activity and endogenous and exogenous cortisol - A systematic review. Psychoneuroendocrinology.

[36] MacLullich AM (2005). Plasma cortisol levels, brain volumes and cognition in healthy elderly men. Psychoneuroendocrinology.

[37] Assis de, G. G. & Gasanov, E. V. BDNF and cortisol integrative system - plasticity vs. degeneration: implications of the val66met polymorphism. *Front Neuroendocrinol* **55**, (2019). 10.1016/j.yfrne.2019.100784 31425696

[38] Hotting K (2016). The effects of acute physical exercise on memory, peripheral BDNF, and cortisol in young adults. Neural Plast.

[39] Numakawa T (2012). The influence of glucocorticoids on neuronal survival and synaptic function. Biomol Concepts.

[40] Raven F (2018). The role of sleep in regulating structural plasticity and synaptic strength: implications for memory and cognitive function. Sleep Med. Rev..

[41] Wadhwa M (2017). Inhibiting the microglia activation improves the spatial memory and adult neurogenesis in rat hippocampus during 48 h of sleep deprivation. J. Neuroinflam..

[42] Mason GM (2021). Sleep and human cognitive development. Sleep Med. Rev..

[43] Gais S, Lucas B, Born J (2006). Sleep after learning aids memory recall. Learn Mem..

[44] Cousins JN, Fernandez G (2019). The impact of sleep deprivation on declarative memory. Prog. Brain Res..

[45] Lu L (2019). The prevalence of sleep disturbances and sleep quality in older chinese adults: a comprehensive meta-analysis. Behav. Sleep Med..

[46] Wang X (2021). The role of perioperative sleep disturbance in postoperative neurocognitive disorders. Nat. Sci. Sleep.

[47] Robinson TN (2009). Postoperative delirium in the elderly: risk factors and outcomes. Ann. Surg..

[48] Shi L (2018). Sleep disturbances increase the risk of dementia: a systematic review and meta-analysis. Sleep Med. Rev..

[49] Huber R, Tononi G, Cirelli C (2007). Exploratory behavior, cortical BDNF expression, and sleep homeostasis. Sleep.

[50] Oh DL (2018). Systematic review of pediatric health outcomes associated with childhood adversity. BMC Pediatr.

[51] April-Sanders A (2021). Childhood adversity and sleep disturbances: longitudinal results in puerto rican children. Int. J. Behav. Med..

[52] Sheehan CM, Li L, Friedman EM (2020). Quantity, timing, and type of childhood adversity and sleep quality in adulthood. Sleep Health.

[53] Giese M (2013). The interplay of stress and sleep impacts BDNF level. PLoS One.

[54] Burgin D (2022). Higher hair cortisol concentrations associated with shorter leukocyte telomere length in high-risk young adults. Sci Rep.

[55] Bernstein DP, Fink L (1998). Childhood trauma questionnaire: a retrospective self-report manual.

[56] WHO, Wellbeing measures in primary health care/The Depcare Project. 1998, Copenhagen: WHO Regional Office for Europe.

[57] Buysse DJ (1989). The pittsburgh sleep quality index: a new instrument for psychiatric practice and research. Psychiatry Res..

[58] Barra, S., et al., Adverse childhood experiences, personality, and crime: distinct associations among a high-risk sample of institutionalized youth. Int. J. Environ. Res. Public Health, 2022. **19**(3). 10.3390/ijerph19031227 PMC8835310 35162246

[59] Jaggi L (2021). Shared residential placement for child welfare and juvenile justice youth: current treatment needs and risk of adult criminal conviction. Child Adolesc Psychiatry Ment Health.

[60] Schmid, M., et al., Abschlussbericht für den Fachausschuss für die Modellversuche und das Bundesamt für Justiz Zusammenfassung der wichtigsten Ergebnisse und Erkenntnisse des Modellversuchs Abklärung und Zielerreichung in stationären Maßnahmen (MAZ). Universitäre Psychiatrische Kliniken Basel und Universitätsklinikum Ulm. Verfügbar unter: https://www.bj. admin.ch/dam/data/bj/sicherheit/smv/modellversuche/evaluationsberichte/maz-schlussbericht-d. pdf, 2011.

[61] d’Huart D (2022). Risikofaktoren für und Stabilität einer Persönlichkeitsstörung vom Jugendalter bis ins junge Erwachsenenalter in einer Hochrisikopopulation. Kindheit und Entwicklung.

[62] d’Huart D (2022). Prevalence and 10-year stability of personality disorders from adolescence to young adulthood in a high-risk sample. Front. Psychiatry.

[63] Schmid M (2022). Misshandlungs- und Vernachlässigungserfahrungen in der Kindheit: Ein Risikofaktor für die soziale Teilhabe ehemals außerfamiliär platzierter junger Erwachsener. Kindheit und Entwicklung.

[64] Seker, S., et al., Mental disorders into adulthood among adolescents placed in residential care: a prospective 10-year follow-up study. European Psychiatry, 2022: p. 1–28. 10.1192/j.eurpsy.2022.30 PMC9280920 35730184

[65] Seker S (2022). Der Verlauf von psychischen Problemen bei fremdplatzierten Kindern und Jugendlichen bis in deren Erwachsenenalter. Kindheit und Entwicklung.

[66] Gao W (2013). Quantitative analysis of steroid hormones in human hair using a column-switching LC-APCI-MS/MS assay. J. Chromatogr. B Analyt. Technol. Biomed. Life Sci..

[67] Schneider E (2023). Effect of short-term, high-dose probiotic supplementation on cognition, related brain functions and BDNF in patients with depression: a secondary analysis of a randomized controlled trial. J. Psychiatry. Neurosci..

[68] Unterholzner, J., et al., Effects of learning and escitalopram administration on serum BDNF levels, a randomised placebo-controlled trial. bioRxiv, 2021.

[69] Wickham H (2016). ggplot2: elegant graphics for data analysis.

[70] Fox, J., John Fox and Sanford Weisberg. An R companion to applied regression, 3rd ed: Sage Publications [Google Scholar]. Published online, 2019.

[71] Giese M (2014). Fast BDNF serum level increase and diurnal BDNF oscillations are associated with therapeutic response after partial sleep deprivation. J. Psychiatr. Res..

[72] Pluchino N (2013). Steroid hormones and BDNF. Neuroscience.

[73] Schauss E (2019). Fostering intrinsic resilience: a neuroscience-informed model of conceptualizing and treating adverse childhood experiences. J. Mental Health Counsel..

[74] van Velzen LS (2016). Effect of childhood maltreatment and brain-derived neurotrophic factor on brain morphology. Soc. Cogn. Affect Neurosci..

[75] Bucker J (2015). Brain-derived neurotrophic factor and inflammatory markers in school-aged children with early trauma. Acta Psychiatr Scand.

[76] Benedetti F (2017). The effect of childhood trauma on serum BDNF in bipolar depression is modulated by the serotonin promoter genotype. Neurosci. Lett..

[77] Theleritis C (2014). Brain derived Neurotropic Factor (BDNF) is associated with childhood abuse but not cognitive domains in first episode psychosis. Schizophr Res..

[78] Vyas N (2023). Systematic review and meta-analysis of the effect of adverse childhood experiences (ACEs) on brain-derived neurotrophic factor (BDNF) levels. Psychoneuroendocrinology.

[79] Greisen MH (2005). Increased adult hippocampal brain-derived neurotrophic factor and normal levels of neurogenesis in maternal separation rats. J. Neurosci. Res..

[80] Zhang, J., et al., IL4-driven microglia modulate stress resilience through BDNF-dependent neurogenesis. Sci. Adv., 2021. **7**(12). 10.1126/sciadv.abb9888 PMC7968840 33731342

[81] Zaletel I, Filipovic D, Puskas N (2017). Hippocampal BDNF in physiological conditions and social isolation. Rev Neurosci.

[82] Monteiro BC (2017). Relationship between brain-derived neurotrofic factor (Bdnf) and sleep on depression: a critical review. Clin. Pract. Epidemiol. Ment. Health.

[83] Kim B (2022). Neighborhoods and sleep health among adults: a systematic review. Sleep Health.

[84] Lee SY (2013). Stress and sleep disturbances in female college students. Am. J. Health Behav..

[85] Morin CM, Rodrigue S, Ivers H (2003). Role of stress, arousal, and coping skills in primary insomnia. Psychosom Med..

[86] Brunborg GS (2011). The relationship between media use in the bedroom, sleep habits and symptoms of insomnia. J. Sleep Res..

[87] Kalmbach DA, Anderson JR, Drake CL (2018). The impact of stress on sleep: Pathogenic sleep reactivity as a vulnerability to insomnia and circadian disorders. J. Sleep Res..

[88] Almojali AI (2017). The prevalence and association of stress with sleep quality among medical students. J. Epidemiol. Glob. Health.

[89] Lai, C.L.J., D.Y.H. Lee, and M.O.Y. Leung, Childhood adversities and salivary cortisol responses to the trier social stress test: a systematic review of studies using the children Trauma Questionnaire (CTQ). Int. J. Environ. Res. Public Health, 2020. **18**(1). 10.3390/ijerph18010029 PMC7793098 33374531

[90] Ohayon MM (2011). Epidemiological overview of sleep disorders in the general population. Sleep Med. Res..

[91] Leger D (2008). An international survey of sleeping problems in the general population. Curr. Med. Res. Opin..

[92] First, M.B., et al., User's guide for the SCID-5-CV Structured Clinical Interview for DSM-5® disorders: Clinical version. 2016: American Psychiatric Publishing, Inc.

[93] Alqahtani, J.S., et al., *Sleep Quality, Insomnia, Anxiety, Fatigue, Stress, Memory and Active Coping during the COVID-19 Pandemic.* Int. J. Environ. Res. Public Health, 2022. **19**(9). 10.3390/ijerph19094940 PMC9104759 35564337

[94] Veqar, Z. and M.E. Hussain, Validity and reliability of insomnia severity index and its correlation with pittsburgh sleep quality index in poor sleepers among Indian university students. Int. J. Adolesc. Med. Health, 2017. **32**(1). 10.1515/ijamh-2016-0090 28063259

[95] Chen B (2001). Increased hippocampal BDNF immunoreactivity in subjects treated with antidepressant medication. Biol. Psychiatry.

[96] Meerlo P, Havekes R, Steiger A (2015). Chronically restricted or disrupted sleep as a causal factor in the development of depression. Curr. Top Behav. Neurosci..

[97] Xie L (2013). Sleep drives metabolite clearance from the adult brain. Science.

[98] Rahmani A (2013). Dehydroepiandrosterone stimulates nerve growth factor and brain derived neurotrophic factor in cortical neurons. Adv. Pharmacol. Sci..

[99] Rusch HL (2015). Improved sleep quality is associated with reductions in depression and PTSD arousal symptoms and increases in IGF-1 concentrations. J. Clin. Sleep Med..

[100] Villegas S, Pecora PJ (2012). Mental health outcomes for adults in family foster care as children: an analysis by ethnicity. Children and Youth Services Rev..

[101] McLaughlin KA (2016). Future directions in childhood adversity and youth psychopathology. J. Clin. Child Adolesc Psychol..

[102] Herzberg MP, Gunnar MR (2020). Early life stress and brain function: Activity and connectivity associated with processing emotion and reward. Neuroimage.

[103] Miguel PM (2019). Early environmental influences on the development of children's brain structure and function. Dev. Med. Child Neurol..

