# Supplementary material for: Interplay between stress, sleep, and BDNF in a high-risk sample of young adults
Source: Sci Rep. 2023 Nov 22;13:20524. doi: 10.1038/s41598-023-47726-0 (PMC10665413; doi:10.1038/s41598-023-47726-0)
Supplement: Supplementary file 1 — Supplementary Information. [file 41598_2023_47726_MOESM1_ESM.pdf]

**Interplay between Stress, Sleep, and BDNF in a High-risk Sample of Young Adults**

Nimmy Varghese[1,2], David Buergin[3,4], Cyril Boonmann[3,54], Christina Stadler[3], Marc Schmid[3], Anne Eckert[1,2#], Eva Unternaehrer[3#*]

1. Research Platform, Molecular & Cognitive Neuroscience, Division of Neurobiology, University of Basel, 4002 Basel, Switzerland
2. Neurobiology Lab for Brain Aging and Mental Health, Psychiatric University Clinics Basel, Medical Faculty, University of Basel, 4002 Basel, Switzerland
3. Child and Adolescent Research Department, University Psychiatric Clinics Basel, University of Basel, Basel, Switzerland
4. Jacobs Center for Productive Youth Development, University of Zurich, Zurich, Switzerland
5. LUMC-Curium – Department of Child of Adolescent Psychiatry, Leiden University Medical Center, Leiden, The Netherlands

\# Shared senior authorship

* Corresponding author

Corresponding Author
Dr. Eva Unternaehrer
Child- and Adolescent Research Department,
Psychiatric University Clinics Basel (UPK),
Wilhelm Klein-Strasse 27
4002 Basel

Tel: +4161 325 80 30
Email: eva.unternaehrer@unibas.ch

# Supplement material

**Supplementary Figure 1**

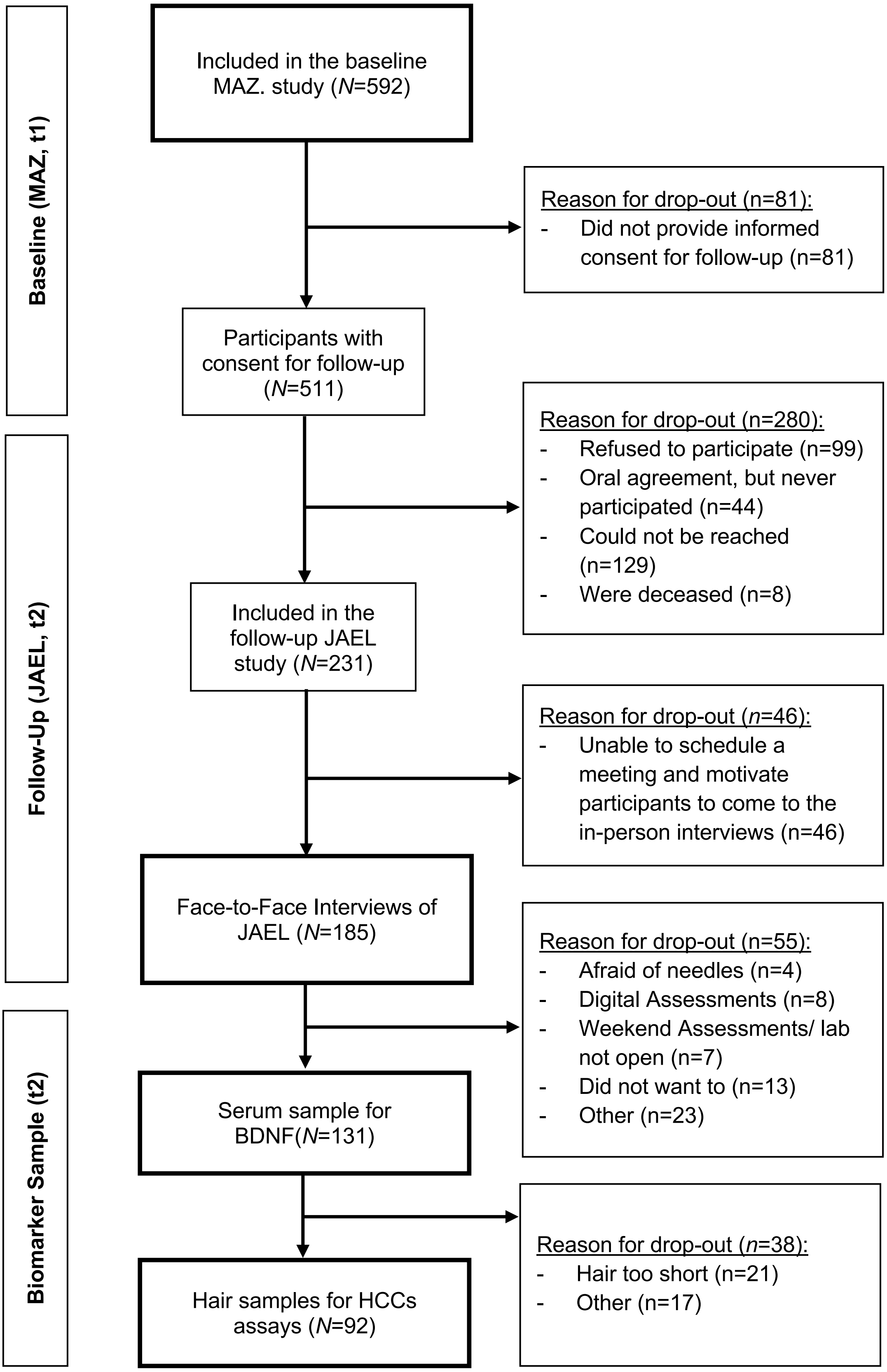

**Supplementary Figure 1.** Flow Chart of Study Participants through the study with final sample sizes included in analyses.

**Supplementary Table 1**

*Correlation matrix of study variables*

| | sBDNF | CTQ | WHO-5 | HCC | PSQI SD |
|---|---|---|---|---|---|
| CTQ (total score)[a] | -0.19* | | | | |
| WHO-5 (total score)[a] | -0.02 | -0.25** | | | |
| HCC (hair cortisol concentration, pg/mg)[a] | 0.15 | -0.06 | 0.07 | | |
| PSQI SD (sleep disturbance score)[a] | 0.09 | 0.31*** | -0.40*** | <0.01 | |
| Age (years)[a] | 0.10 | -0.09 | 0.07 | -0.18 | -0.04 |
| BMI (kg/m2)[a] | -0.02 | -0.03 | -0.02 | 0.04 | 0.03 |
| Sex[b] | -0.08 | 0.36*** | -0.22* | 0.16 | 0.25** |
| SES[b] | 0.10 | 0.18 | -0.16 | 0.01 | 0.21* |
| Migration status[b] | -0.15 | -0.04 | -0.16 | 0.06 | 0.18* |
| Medication status[b] | 0.08 | 0.17 | -0.13 | -0.06 | 0.25** |
| Current smoking status[b] | 0.19 | 0.04 | -0.10 | -0.01 | 0.07 |
| Number of cigarettes[a] | 0.09 | 0.10 | 0.08 | 0.13 | 0.10 |
| Eating 2h prior to blood withdrawal[b] | 0.18 | 0.08 | -0.17 | 0.12 | 0.14 |
| Caffeine intake 2h prior to blood withdrawal[b] | 0.06 | 0.10 | 0.01 | 0.07 | -0.03 |
| Alcohol intake 2h prior to blood withdrawal[b] | -0.10 | 0.08 | 0.08 | 0.05 | -0.06 |
| Physical activity 2h prior to blood withdrawal[b] | 0.01 | 0.01 | 0.12 | 0.05 | -0.07 |

Note. BMI = Body Mass Index; CTQ = Childhood Trauma Questionnaire; HCC= hair cortisol concentration; PSQI SD = Pittsburgh Sleep Quality Inventory Sleep Disturbances Score; sBDNF = serum brain-derived neurotrophic factor; SES = socioeconomic status; WHO-5 = World Health Organization Well-being Questionnaire; [a]Spearmans correlation coefficient; [b]Pearsons correlation coefficient; *p<.05; **p<.01; ***p<.001
